# IGF-1 Induction by Acylated Steryl β-Glucosides Found in a Pre-Germinated Brown Rice Diet Reduces Oxidative Stress in Streptozotocin-Induced Diabetes

**DOI:** 10.1371/journal.pone.0028693

**Published:** 2011-12-14

**Authors:** Seigo Usuki, Ying-Ying Tsai, Keiko Morikawa, Shota Nonaka, Yasuhide Okuhara, Mitsuo Kise, Robert K. Yu

**Affiliations:** 1 Institute of Molecular Medicine and Genetics, Georgia Health Sciences University, Augusta, Georgia, United States of America; 2 FANCL Research Institute, FANCL Corporation, Yokohama, Japan; Boston University, United States of America

## Abstract

**Background:**

The pathology of diabetic neuropathy involves oxidative stress on pancreatic β-cells, and is related to decreased levels of Insulin-like growth factor 1 (IGF-1). Acylated steryl β-glucoside (PR-ASG) found in pre-germiated brown rice is a bioactive substance exhibiting properties that enhance activity of homocysteine-thiolactonase (HTase), reducing oxidative stress in diabetic neuropathy. The biological importance of PR-ASG in pancreatic β-cells remains unknown.

Here we examined the effects of PR-ASG on IGF-1 and glucose metabolism in β-cells exposed to oxidative stress.

**Methodology/Principal Findings:**

In the present study, a pre-germinated brown rice (PR)-diet was tested in streptozotocin (STZ)-induced diabetic rats. Compared with diabetic rats fed control diets, the PR-diet fed rats showed an improvement of serum metabolic and neurophysiological parameters. In addition, IGF-1 levels were found to be increased in the serum, liver, and pancreas of diabetic rats fed the PR-diet. The increased IGF-1 level in the pancreas led us to hypothesize that PR-ASG is protective for islet β-cells against the extensive injury of advanced or severe diabetes. Thus we examined PR-ASG to determine whether it showed anti-apoptotic, pro-proliferative effects on the insulin-secreting β-cells line, INS-1; and additionally, whether PR-ASG stimulated IGF-1 autocrine secretion/IGF-1-dependent glucose metabolism. We have demonstrated for the first time that PR-ASG increases IGF-1 production and secretion from pancreatic β-cells.

**Conclusion/Significance:**

These findings suggest that PR-ASG may affect pancreatic β-cells through the activation of an IGF-1-dependent mechanism in the diabetic condition. Thus, intake of pre-germinated brown rice may have a beneficial effect in the treatment of diabetes, in particular diabetic neuropathy.

## Introduction

Dietary intake of pre-germinated brown rice (PR) (*Hatsuga genmai*) [1] has widely been found useful for alleviating symptoms of both diabetes and pre-diabetes. Previously we demonstrated that intake of a PR-containing diet decreased the severity/frequency of hyperglycemia in streptozotocin (STZ)-induced diabetic rats [2]. Later it was demonstrated that the beneficial effects of the PR-diet were due to the germination process, which was considered contributory to development of the intrinsic potential to elevate the activities of protective enzymes that respond to oxidative stress in diabetic neuropathy [3]. One such known stress is an increase in homocysteine (Hcy), which is elevated in diabetes [3], and undergoes intra-molecular thiolactone formation.

Acylated steryl β-glucoside (ASG) is a glycolipid that is ubiquitously distributed in edible plant sources [4]. We revealed that germination of brown rice led to *de novo* production of a special lipid ingredient, PR-derived ASG (PR-ASG), as purified from the bran of PR [5]. PR-ASG was found to be a bioactive substance that enhances the activities of the enzyme Hcy-thiolactonase (HTase) to decompose Hcy-thiolactone and decrease severity of oxidative stress and diabetes. This enhancing activity has thus far been demonstrable only for PR-ASG and has not been shown for any other ASGs contained in plant seeds.

It is unclear how PR-ASG is related to the anti-oxidative activity of the PR-diet. PR-ASG may possess unknown and protective activity for diabetes beyond the oxidation defense provided by activation of HTase. It is also unclear whether PR-ASG may for example up-regulate the β-cells self-anti-apoptosis machinery, enabling β-cells to rescue themselves from oxidative stress and cell death by diabetes. Oxidative stress occurs secondary to an increase in the level of Reactive oxgen species (ROS), which is controlled primarily by the defense system against oxidative stress in β-cells. There is a critical balance between endogenous ROS generation and antioxidant defense in the β-cells. The overall effect of the antioxidant system depends on the intracellular balance between these antioxidant enzymes [6]. The mechanism for maintaining that enzymatic balance involves glucose-6-phosphate dehydrogenase (G6PD). The function of G6PD is to maintain the cellular ratio of NADPH/NADP and up-regulate its own activity in the pentose phosphate pathway relevant to the cell apoptotic response to ROS [7], [8].

Glucose is implicated as being a regulatory molecule for inducing β-cells to induce secretion of insulin and insulin-like growth factor 1 (IGF-1). It is known that this glucose-dependent IGF-1 activation system is closely coupled to glucose metabolism including the glycolytic pathway, and the pentose phosphate pathway [8], [9], [10]. For example, activation of the glucose-dependent IGF-1 activation system subsequently enhances the glycolytic pathway for cell proliferation [8]. It is well known that each of these pathways can be blocked by specific inhibitors: 6-aminonicotinamide (6-AN) for the pentose phosphate pathway, and 2-deoxyglucose (2-DG) for the glycolytic pathway [11], [12].

In the present study we focused on IGF-1 in pancreatic islet β-cells, since IGF-1 activity also is intimately related to development of diabetic neuropathy. The relationship of diabetic neuropathy to various growth factors has been extensively studied [13]. In particular, IGF-1 is known to be decreased in serum of rats with diabetic neuropathy [14], [15].

In order to examine the relationship between PR-ASG and IGF-1, we determined how PR-ASG affects IGF-1 levels of serum, pancreas, and liver in STZ-diabetes rats fed PR diet. Subsequently we used an islet β-cell line (INS-1) to examine replication and apoptosis of β-cells, which are involved in hyperglycemia-induced oxidative stress in diabetes [10], [14]. Our data suggests that PR-ASG enhances IGF-1 production in STZ-diabetic rat islet β-cells and INS-1 cells by the same mechanism. To understand this action of PR-ASG, we analyzed the effects that it had on two systems for defense against oxidative stress. The first system is the IGF-1-related pentose phosphate/glycolytic pathways; this system was analyzed with the specific inhibitors 6-AN and 2-DG. The second is the PR-ASG-sensitive HTase-related anti-oxidation system.

## Materials and Methods

### Ethics statements

The use of these animals was approved by Georgia Health Sciences University's Institutional Animal Care and Use Committee (protocol# 06-08-828).

### Experimental diets

Pre-germinated brown rice (PR) and white rice (WR), both of the *Japonica* species, were used in the study. PR was prepared at 25–30% water content to induce germination and dried to 15% according to a patented procedure (Patent No. 3738025, Jp, November 4, 2005).

PR and WR were manufactured as powdered feed by Harlan Teklad (Madison, WI). The control diet (AIN93G) was composed of cornstarch [39.7% (w/w)], α-cornstarch [13.2% (w/w)], casein [20.0% (w/w)], L-cysteine [0.3% (w/w)], sucrose [10% (w/w)], soybean oil [7.0% (w/w)], cellulose powder [5.0% (w/w)], mineral mix [3.5% (w/w)], vitamin mix [1.0% (w/w)], choline bicitrates [0.25% (w/w)], and butylhydroquinone [0.0014% (w/w)]. PR and WR diets were produced by replacing cornstarch and α-cornstarch with the respective powders as a source of carbohydrates. The energy content range was 370.0 to 390.0 kcal/100 g diet, with no total energy difference between the experimental diets.

### STZ-induced diabetic rats and experimental diets

Male Wistar rats weighing 120–140 g received intra-peritoneal injections of STZ (65 mg/kg in 100 mM sodium citrate buffer, pH 4.5) and were maintained on control diet (AIN93G) for 2 weeks. Two animals were housed per cage in a controlled environment. All animals were allowed free access to water and feed.

Only those animals whose blood glucose levels were >400 mg/dl were diagnosed as diabetic. The diabetic rats (n = 24) were divided into three diet-feeding groups: AIN93G (n = 8), WR (n = 8), and PR (n = 8). Non- diabetic, control rats (n = 18) were also divided into the same three diet-feeding groups: AIN93G (n = 6), WR (n = 6), and PR (n = 6). The substitution of control diet with experimental diet was performed at 2 weeks after STZ injection and continued for 3 weeks. Food consumption was measured by weighing the feeding containers daily. Beginning 1 week after STZ injection, blood samples were obtained at predetermined time points (0, 1, 2, 3, 4, and 5 weeks) from the same rats following a 15-hour starvation period. Blood was obtained by pricking the tip of the rat tail and collecting a drop (about 0.06 ml) of blood. The glucose levels in these blood samples were measured using a strip-operated blood glucose meter (Accu-Chek Advantage Blood Glucose Meter, Roche Diagnostics, Indianapolis, IN). When the blood glucose level was higher than the range of the instrumental system, the serum glucose level was assessed by Glucose (GO) Assay Kit (GAGO-20, Sigma, St. Louis, MO). Three weeks after initiation of the experimental diets, nerve conduction studies were performed in the rats' tails to assess electrophysiological function, after which the rats were anesthetized. Whole blood was obtained from each rat by cardiocentesis at the endpoint of the experiment, and serum samples were obtained for biochemical analyses, as described below.

### NCV measurements

Nerve-conduction velocities (NCVs, m/sec) were assessed in the rat tail nerve using a Nicolet Viking Quest EMG machine (Neurocaregroup, Madison, WI) according to the modified procedure of Anderson *et al.* [16]. In summary, the tail nerves were stimulated using external digital ring electrodes with flexible wires (Medtronic Functional Diagnostics, Skovlunde, Denmark) in place of needle electrodes [3]. The electrodes were attached to the tail segments 5 cm proximal and 2 cm distal to the recording position (7-cm distal from the joint of the rat's tail). Blood was then collected from the distant tip of the tail that was non-interactive with NCV measurement. Electrical stimulation of each rat generated four different waves, from which NCV was evaluated under the condition that every wave showed a reproducible latency and amplitude as the voltage of the stimulation was increased. During each measurement, the surface temperature of the rat's tail was maintained (34–35°C). Four nerve conduction waves were measured per animal; the NCV values were corrected for monitor variations in temperature and expressed as an average value.

### Serum metabolic parameters in animal experiments

Homocysteine (Hcy), high density lipoprotein-cholesterol (HDL-C), and low density lipoprotein-cholesterol (LDL-C) were respectively measured by the following commercial assay kits from Diazyme Laboratories (San Diego, CA): HDL-Cholesterol Test Kit; LDL-Cholesterol Test Kit; Homocysteine Assay Kit. HTase activity was measured using an Alfresa Auto HTLase assay kit from Alfresa Pharma Corp. (Osaka, Japan) [17].

### Measurement of IGF-1 and insulin

For IGF-1 assay, liver and pancreas tissue from each rat was rinsed with phosphate buffered saline (PBS), and homogenized in PBS solution (20% w/v) and stored overnight at −40°C. After two freeze-thaw cycles, the homogenates were centrifuged for 5 min at 5,000×g. The tissue supernatants were immediately collected and frozen before use in IGF-1 assay. Serum samples also were immediately collected and frozen before use for the IGF-1 and insulin assay. An immunoassay kit that is able to recognize rat/mouse IGF-1 (Quantikine®, R&D Sysetm, Inc., Minneapolis, MN) was used to quantify IGF-1 in serum and tissue supernatant from each rat. Serum insulin was measured by suing Rat/Mouse Insulin ELISA kit (Millipore, Billerica, MA).

### Purification of PR-ASG for in vitro experiments

Using the procedure detailed by Folch [18], a total lipid fraction was prepared from bran of PR by successive extraction of 5 g samples with 30 mL of chloroform-methanol [1∶1(v/v)] and 20 mL of chloroform-methanol [2∶1(v/v)]. The lipid extracts were combined, evaporated, and subjected to silica gel column chromatography (Iatrobeads, 6RS-8060, Iatron, Tokyo, Japan) according to a previously described method [5].

PR-ASG was analyzed by thin-layer chromatography on a high performance thin layer chromatographic plate (HPTLC) that was developed using a solvent system of chloroform-methanol-water (65∶35∶8; v/v/v). It was judged to be homogeneous, as revealed by a single spot upon visualization using the orcinol-sulfuric acid reagent.

Subsequently, the activity of the purified PR-ASG in HDL fractions from non-diabetic male Wistar rats was tested for HTase using an Alfresa auto HTLase assay kit (Alfresa Pharma Corp., Osaka, Japan).

For cell experiment PR-ASG was dissolved in RPMI 1640 medium containing 0.1% bovine serum albumin (fatty acid-free and low endotoxin, Sigma, St. Louis, MO, USA).

### Cells

The parental INS-1 cell line was established from a rat insulinoma by Asfari et al [19]. Cells were maintained at 37°C in an atmosphere of 5% CO_2_ and 95% air with RPMI 1640 medium containing 10% fetal bovine serum supplemented with 10 mM HEPES, 1 mM sodium pyuruvate, 50 µM 2′-mercaptoethanol, penicillin (100 units/mL), and streptomycin (100 µg/mL). Cells were subcultured to 70–80% confluence [19].

### Cell proliferation/toxicity and released IGF-1/insulin levels

Cells (5×10^4^ in 200 µL per well) were seeded into a 96-well micro plates coated with poly-L-ornithine (0.001%) in 10% serum-RPMI 1640 medium. After 24 hours incubation, the cells were washed twice with serum-free medium, and then exposed for 1 hr to 1 mM STZ in the serum-free medium, either in the presence or absence of PR-ASG (0–400 µg/mL). Subsequently, the cells were washed twice more, and cultured for 2 days at 37°C with serum-free medium containing between 0 and 400 µg/mL PR-ASG. Following culture, cell viability testing was performed by using cell counting kit-8 (CCK-8, Dojindo, MD, USA); and an IGF-1 immunoassay kit (Quantikine®, R&D Sysetm, Inc., Minneapolis, MN) was used to measure IGF-1 released into the cultured medium. In order to induce insulin secretion, cells were washed Krebs-Ringer buffer pH 7.4 (119 mM NaCl, 4.75 mM KCl, 2.54 mM CaCl_2_, 1.2 mM MgSO_4_, 1.2 mM KH_2_PO_4_, 5 mM NaHCO_3_, 20 mM HEPES, pH 7.4) and exposed to low glucose (2 mM) buffer medium and high glucose (12 mM) buffer medium for 1 hr. Insulin determination was performed on the incubation medium using Rat/Mouse Insulin ELISA kit (Millipore, Billerica, MA).

### TUNEL assay and nuclear staining of INS-1 cells

Cells were cultured on glass coverslips, and then exposed to 1 mM STZ in serum-free medium for 1 hour. The cultured cells were then divided into serum and ASG groups, and respectively supplemented with either serum (0 and 10%) or PR-ASG (4 and 400 µg/mL). Subsequently, cells were cultured for 2 days in fresh serum-free medium and supplemented with either serum (0 and 10%) or PR-ASGs (4 and 400 µg/mL), respectively. After pre-washing 3 times with PBS, cells were fixed with 4% paraformaldehyde/PBS solution. They were then permeabilized at 37°C for 1 hour with 100 µL TUNEL mixture (Roche, Mannheim, Germany). The cells were again washed with PBS, followed by staining with 4′, 6′-dianidino-2′-phenylindole diHCl (DAPI; 1 µg/mL; Sigma) in the dark for 30 min. After a final washing of the cells, the coverslips were mounted on glass slides and examined under a fluorescent microscope (Nikon Eclipe TE300, Nikon Instruments, Melville, NY).

### mRNA expression of IGF-1 in INS-1 cells

Cells (1.0×10^7^) were seeded in 10 mL of 10% serum-RPMI 1640 medium on a 100-mm dish that had been precoated with poly-L-ornithine (0.001%). One day later, the cells were washed twice with serum-free medium and pre-incubated at 37°C in this medium supplemented with 10% serum or varying quantities of PR-ASG (4 and 8 µg/mL, respectively). Two days later, the cells were washed with 2 mL of PBS twice and then harvested for RNA preparation.

Total cellular RNA was isolated using NucleoSpin® RNA II Kit (Marcherey-nagel, Düren, Germany) and quantitated at wavelength 260 nm on a spectrophotometer (Beckman Coulter Inc., Fullerton, CA). The isolated total RNA was then converted to cDNA using Superscript III First-strand Synthesis System (Invitrogen, CA), following the protocol supplied by the manufacturer. The PCR conditions were 94°C denaturation for 20 sec, followed by 60°C annealing for 20 sec, and then 72°C extension for 40 sec for 26–38 cycles. Gene expression was described as positive or negative expression in relation to the ‘housekeeping’ β-actin gene. The PCR products were analyzed by 2% agarose gel electrophoresis. The sequences of the primers used for the gel were as follows: IGF-1 sense (5′- CTGCTTGCTCACCTTTACCA -3′), IGF-1 antisense (5′- GCTCAAGCAGCAAAGGATCT -3′), β-actin sense (5′- ACGGTCAGGTCATCACTATCG -3′), and β-actin antisense (5′- GCTCAAGCAGCAAAGGATCT -3′).

### Glucose utilization in INS-1 cells

Cells (5×10^4^ in 200 µL per well) were seeded into 96-well micro plates coated with poly-L-ornithine (0.001%) in RPMI 1640 medium containing 10% serum. One day later, the cells were washed twice with serum-free medium, followed by exposure for 1 hr to either 1 mM STZ or no STZ, in serum-free medium containing either 0 or 4 µg/mL PR-ASG. Subsequently, cells were pre-washed twice with Krebs Ringer buffer (pH 7.4). They were then exposed for 1 day to 0.1 mM glucose and 0–300 ng/mL IGF-1(Recombinant Rat Insulin-Like Growth Factor 1, #CR117; Cell Sciences Canton, MA) in Krebs Ringer buffer, containing 0–10 mM 2-deoxy-glucose (2-DG; Sigma) and either 0 or 4 µg/mL PR-ASG. After treatment, the supernatant of the cells was examined for glucose utilization using Glucose (GO) Assay Kit (GAGO-20, Sigma). . This glucose utilization was less than 1.0 nmol/hr/10^6^ cells at zero concentration of IGF-1. A percent value denotes that 100% is equal to 16.7 nmol/hr per 10^6^ cells. The cell numbers were normalized using values measured during collection of the supernatants.

### Glucose-6-phosphate dehydrogenase (G6PD) activity in INS-1 cells

Cells (2.5×10^5^ in 1 mL per well) were seeded into 12-well plates coated with poly-L-ornithine (0.001%) in 10% serum-RPMI 1640 medium. One day later, the cells were pre-washed with serum-free medium, and treated by the following procedure: 1) the cells were exposed for 24 hours to the presence (4 µg/mL) or the absence (0 µg/mL) of PR-ASG in either the RPMI medium containing 0–100 µM 6-AN or the RPMI medium containing no folic acid and between 0–50 mM Hcy; and 2) the cells were exposed for 1 hr to either 1 mM STZ or no STZ, in serum-free medium containing either 0 or 4 µg/mL PR-ASG, followed by pre-washing with fresh medium and treated for 30 min at 37°C with serum-free RPMI-1640 medium containing 0–32 mM glucose and 4 µg/mL PR-ASG.

In both of the above procedures, cells were harvested and sonicated for 1 min in 50 mM Tris-HCl buffer (pH 7.2). The supernatants were collected in micro-centrifuge tubes and normalized based on the relationship between protein amount and cell number. The enzyme reaction mixture was composed of 10 µL of the supernatant and 90 µL of the following solution: 50 mM Tris-HCl buffer (pH 7.2) containing 2.5 mM glucose-6-phosphate; 0.2 mM NADP; 0.2 mM 1-methoxy-phenazine methosulfate; and 5 mM WST-8; tetrazolium salts, Sigma). This reaction mixture was incubated for 30 min at 37°C, followed by colorimetric measurement at 450 nm with a reference at 595 nm.

### Measurement of total ROS in INS-1 cells

Total ROS level was detemined using 2′, 7′-dichlorodihydrofluorescein diacetate (DCF-DA) (Sigma). Cells were seeded into 96-well microtiter plates (white plate with clear bottom, Corning) in RPMI 1640 medium containing 10% serum. One day later, the cells were pre-washed twice, and treated for 1 hour with 1 mM STZ. Subsequently, cells were pre-washed with PBS, and incubated with DCF-DA (1.0 µM) in darkness for 10 min. DCF-DA green fluorescence was assessed under a fluorescent microscope (Nikon Eclipe TE300, Nikon Instruments, Melville, NY) and quantitated by a Perkin-Elmer Victor V multilabel plate reader with excitation/emission filters set at 490 and 535 nm.

### Measurement of HTase activity in INS-1 cells

Cells (2.5×10^5^ in 1 mL of 10% serum-containing RPMI medium per well) were cultured in12-well plates. One day later, the cells were exposed for 1 hr to 1 mM STZ with either 0 or 4 µg/mL of PR-ASG, in RPMI 1640 medium, containing 1.0 µg/mL low density lipoprotein (LDL), prepared from rat by our method as previously reported [3]. Subsequently, the cells were washed and treated for 24 hr in serum-free RPMI-1640 medium containing either 0 or 4 µg/mL of PR-ASG. Cells were then harvested and sonicated for 1 min in PBS buffer, and the supernatants were collected in micro-centrifuge tubes. The cellular supernatant was added to an Alfresa auto HTLase assay kit (Alfresa Pharma Corp., Osaka, Japan). The initial rate of hydrolysis was determined spectrophotometrically by monitoring absorbance at 450 nm both 0 and 10 min after substrate addition. One unit of HTase activity is equal to the amount of 1 nmol HT that is hydrolyzed in 1 minute by 1 mg of protein.

### Statistical analysis

Statistical analyses were performed using the GraphPad Prism 5.0 software package (GraphPad, San Diego, CA, U. S. A.). The various parameters evaluated in our animal experiments were analyzed using the one-way ANOVA, followed by Tukey's multiple comparison test and Dunnet's test (Table 1). The influences of PR-ASG in cell experiments was analyzed for statistical differences using either one-way ANOVA followed by Tukey's and Dunnet's test, or two-way ANOVA followed by Bonferroni's post-test.

**Table 1:** Final body weights, blood glucose concentrations, and serum parameters in non-diabetic and diabetic rats (3 weeks).

Treatment	Non-diabetic			Diabetic		
Diet	AIN93G	WR	PR	AIN93G	WR	PR
Numbers of animals	6	6	6	8	8	8
Weight (g)	370.1±10.5	362.3±11.4	356.2±11.4	174.5±15.1	182.2±17.8	213.8±21.8
Blood Glucose(mg/dl)	107.3±19.0	106.2±20.1	112.3±12.1	457.3±38.1	425±32.1	301.2±32.7**
NCV (m/s)	50.1±1.2	48.9±1.2	49.8±1.3	35.1±1.5	34.9±2.0	45.0±1.3***
Hcy (uM)	5.1±0.5	5.1±0.2	5.1±0.3	2.4±0.6	2.2±0.4	4.7±0.5**
HTase (nml/mg/min)	8.3±0.5	8.6±0.2	9.1±0.6	3.9±0.8	4.3±0.9	7.9±0.8**
HDL-C (mg/dL)	45.4±3.1	44.1±2.9	43.9±2.3	21.8±2.4	22.9±4.1	39.2±3.1**
LDL-C (mg/dL)	81.5±5.2	80.0±5.8	77.7±4.1	120.1±3.2	123.1±2.9	101.3±3.8**
Serum IGF-1 (ng/mL)	961.5±87.6	920.9±75.5	978.7±54.9	160.9.1±82.1	167.8±89.1	607.3±98.9**
Pancreas IGF-1 (ng/g wet tissue)	152.4±50.2	142.2±35.2	155.8±25.8	51.1±22.0	65.3±25.1	120.1±32.0**
Liver IGF-1 (ng/g wet tissue)	231.9±13.9	240.5±15.6	244.1±19.0	85.9±17.6	92.0±16.7	165.1±15.9*
Serum insulin (ng/mL)	4.9±0.08	5.1±0.07	5.2±0.08	0.3±0.08	0.35±0.06	0.62±0.07*

One-way ANOVA was performed to analyze the variation between 3 groups of non-diabetic or diabetic treatment. The data were then analyzed using Tukey's multiples comparison test (if parametric). Values were expressed as means±SEM. Differences between the PR-treated group and other diet groups were analyzed by Dunnet's multiple comparison test (*p<0.05, **p<0.01, ***p<0.001).

## Results

### PR-ASG

The current lot of PR-ASG used in our cell experiment was tested for HTase activity according to a commercial assay kit for HTase (Alfresa Auto HTLase; Alfresa, Osaka, Japan). HTase activity was quantitated by measuring hydrolysis of the substrate, γ-thiobutyrolactone, along with either 100 µg of rat HDL fraction in the presence of 1.0 µg of PR-ASG, or 100 µg of rat HDL fraction in the absence of PR-ASG. Purified PR-ASG clearly showed a stimulatory effect on HTase activity, at 0.01 to 1.0 µg. This increase with addition of PR-ASG reflects up to 160% of enhancement (16.4±0.52 nmol/min) as compared with HTase activity in the absence of PR-ASG (10.4±0.32 nmol/min). For 6 different measurements of HTase activity level, the resulting values were presented as mean±SEM and showed a statistical difference (p<0.01).

### Body weight and blood-glucose level in rats

An animal experiment was performed on STZ-induced diabetic rats in order to examine the effect of PR-diet. During the 3-week experimental feeding period the rats' food consumption rate (g per day) showed no difference in food intake between normal and diabetic rats.

No difference in weight gain was observed in normal rats fed any of 3 varying diets (WR-, PR-, and AIN93G). Nor did diet type appear to effect weight gain in the diabetic rats; however, overall weight gain was suppressed in the diabetic rat population (Table 1), as we have previously reported [3]. This is considered to be a well-established phenomenon observed in insulin-deficient rats after STZ treatment [20].

The blood-glucose levels of the non-diabetic rats on all 3 diets (WR, PR, and AIN93G) were within the normal range during the experimental period (Table 1). In contrast, the blood-glucose levels of the diabetic rats were elevated; however, the blood glucose levels of diabetic rats on the PR diet were significantly lower than those fed the AIN93G and WR diets (*p*<0.01, Dunnet's test).

### Neurophysiological parameter and serum metabolic parameter and in rats

To evaluate the effect of the PR-diet on diabetic neuropathy, serum metabolic parameters and nerve conduction velocity (NCV) were measured. The effect of the various diets on NCV is shown in Table 1, expressed as means ± SEM. Diabetic rats fed all 3 diets showed significantly decreased NCVs when compared with the NCVs of the non-diabetic rats. Within the diabetic rat population, however, NCVs in PR-diet treated rats were significantly improved as compared with NCVs in rats fed AIN93G and WR-diets.

Table 1 also shows serum Hcy level and HTase activity for all groups of rats. Control diet (AIN93G) feeding in STZ-treated rats showed abnormality of serum parameters specific to diabetes; but non-diabetic rats showed normality of serum parameters. There was no difference of serum parameters between control diet and two experimental diets in non-diabetic rats. In the diabetic rat population, on the other hand, when those fed the AIN93G and WR diets were compared to those fed the PR-diet, the former group showed a statistically significant increase in both Hcy level and HTase activity(**p<0.01). Because the diabetic rats were accompanied by an increase in creatinine level, the decreased Hcy level may be attributed to renal insufficiency, such as occurs in diabetic nephropathy (data not shown). However, we found that the Hcy level could normalize in diabetic rats fed the PR diet.

Serum cholesterol-balance resulted in an increase in LDL-C and a decrease in HDL-C in diabetic rats fed the AIN93G and WR diets, compared to the non-diabetic rats. Contrary to the diabetic rats fed the AIN93G and WR diets, the diabetic rats fed the PR diet showed statistically significant normalization of LDL-C and HDL-C levels.

### IGF-1 and insulin levels in rats

Serum insulin levels were significantly reduced in diabetic rats treated with AIN93G diet compared with non-diabetic rats (Table 1). However, the PR-diet-treated diabetic rats showed increased insulin levels compared with diabetic rats treated with AIN93G- and WR-diets (**p<0.01). IGF-1 levels in rat serum, pancreas and liver samples are shown in Table 1. All 3 control (non-diabetic) groups had IGF-1 levels as follows: serum levels around 950 ng/mL; pancreas and liver levels around 150 and 238 ng/g tissue, respectively. In contrast, diabetic rats fed the AIN93G and WR diets showed a marked decrease in serum IGF-1 levels (160.9 and 167.8 ng/mL), liver (85.9 and 92.0 ng/g tissue), and pancreas (51.1 and 65.3 ng/g tissue). As compared with AIN93G diet, diabetic rats fed the PR diet showed a statistically significant rebound in IGF-1 levels in serum (from 160.9 to 607.3 ng/mL, **p<0.01), pancreas (from 51.1 to 120.1 ng/g tissue, **p<0.01), and liver (from 85.9 to 165.1 ng/g tissue, *p<0.01). Above all, remarkable was the IGF-1 enhancement in the pancreas. In contrast non-diabetic rats on the PR-diet did not show any significant rebound in the pancreas IGF-1 level as compared with AIN93G and WR diets.

### Cell toxicity and apoptosis in INS-1 cells

**Figure 1:**
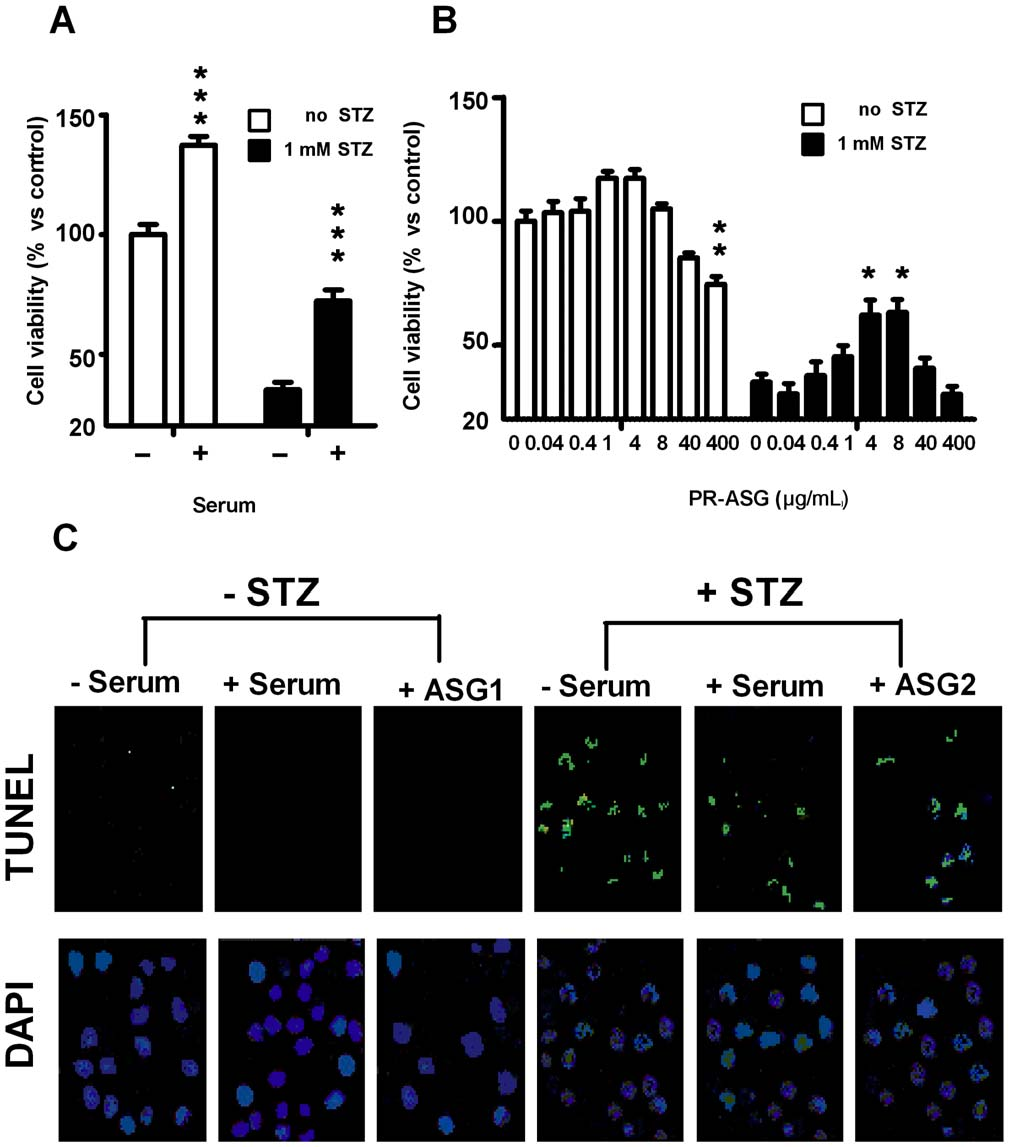
Effect of serum and PR-ASG on cell viability and cell apoptosis under STZ treatment. A) Cells were exposed either to 1 mM STZ (▪) or to no STZ (□) in serum-free RPMI 1640 medium for 1 hr, followed by 2-day culturing either in serum-free (−) or in 10% serum (+) medium. The treated cells were tested for viability using WST-8 assay. B) Cells were exposed for 1 hr either to 1 mM STZ (▪) or to no STZ (□) in serum –free RPM 1640 medium supplemented with increasing amounts of PR-ASG (0–400 µg/mL). This was followed by 2 days of culturing in fresh serum-free medium, again supplemented with increasing amounts of PR-ASG (0–400 µg/mL). The treated cells were tested for viability using WST-8 assay. Values are expressed mean ± SEM, with n = 6 individual experiments. Data were analyzed by one-way ANOVA followed by Tukey's test and then Dunnet's test. The multiple asterisks denote a statistically significant difference between the first column (non-shaded or shaded), as compared with each of the other columns (non-shaded or shaded, respectively) (*p<0.05, **p<0.01, and ***p<0.001). C) Cells were exposed to STZ, serum, and PR-ASG. They were then examined by TUNEL assay and nuclear staining (DAPI) with parameters described as follows: −STZ (no STZ), +STZ(1 mM STZ), −Serum (serum free), +Serum (10% serum), ASG1(400 µg/mL PR-ASG), and ASG2(4 µg/mL PR-ASG).

To determine the effect of PR-ASG on cell growth and death of β-cells, INS-1 cells were cultured in medium with 10% serum. These cells showed better viability, increasing in number by about 140% as compared with those grown in serum-free medium (Fig. 1A). On the other hand, pre-treatment with STZ decreased cell viability remarkably due to the toxicity of STZ. However, 10% serum was found to be protective against STZ toxicity and improved cell viability up to 55.1% of that of control. The decrease in the number of viable, STZ treated cells was analyzed using a cell apoptosis assay with TUNEL, and nuclear staining with DAPI (Fig. 1C). We found that STZ induced INS-1 cell apoptosis, but that 10% serum was protected against said apoptosis.

PR-ASG (at 1 and 4 µg/mL) enhanced INS-1 cell proliferation, although we did not find statistically significant differences from the control group (Fig. 1B). A high concentration of PR-ASG (400 µg/mL) showed some toxicity, by causing cell detachment from the plate as shown in picture ASG1 of Fig. 1C. Contrarily, a low concentration of PR-ASG (4 and 8 µg/mL) was protective against cell apoptosis secondary to STZ treatment (Fig. 1B and C).

### IGF-1 release into the culture medium and mRNA expression of IGF-1

**Figure 2:**
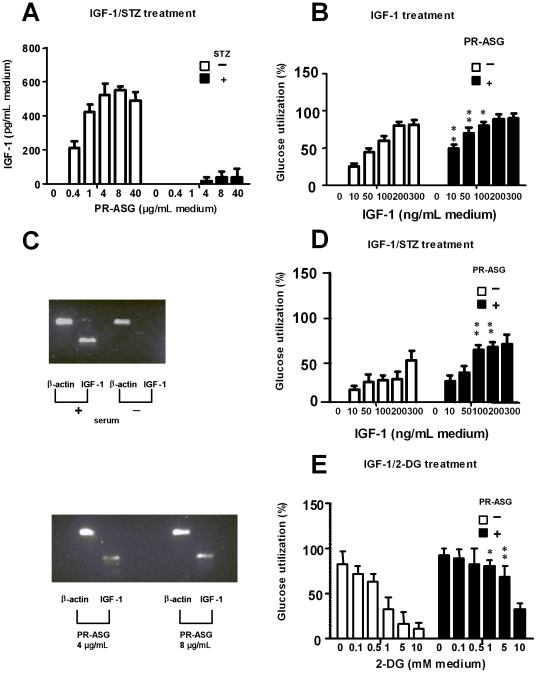
Effect of PR-ASG treatment on IGF-1 secretion, mRNA production, and glucose utilization in INS-1 cells. A) Cells were exposed for 1 hr either to 1 mM STZ (▪) or to no STZ (□) in serum-free RPMI 1640 medium supplemented with increasing amounts of PR-ASG (0–40 µg/mL), followed by 2 days of culturing in fresh serum-free medium supplemented with increasing amounts of PR-ASG (0–40 µg/mL). Subsequently, IGF-1 in the cell-conditioned medium was measured using IGF-1 assay kit. Values are expressed as mean ± SEM, n = 6 individual experiments. B) Cells were pre-washed with Krebs Ringer buffer. They were then cultured for one day with Krebs Ringer buffer containing 0.1 mM glucose supplemented with increasing amount of IGF-1 (0–300 ng/mL), and either 4 µg/mL (▪) or no (□) PR-ASG. Glucose utilization was examined in the supernatants of these cells using a glucose assay kit. This glucose utilization was less than 1.0 nmol/hr/10^6^ cells at zero concentration of IGF-1. A percent value denotes that 100% is equal to 16.7 nmol/hr per 10^6^ cells. C) Cells (1.0×10^7^) were seeded in 10% serum- containing RPMI 1640 medium in a 100-mm dish that had been precoated with 0.001% poly-L-ornithine. One day later, the cells were pre-washed twice using RPMI medium, followed by exposure either to 10% serum or 0, 4, and 8 µg/mL of PR-ASG in serum-free medium. Two days later, RT-PCR was performed on the harvested cells as described in the Materials and Methods section. D) Cells were exposed for 1 hr to 1 mM STZ in serum-free medium containing either 4 µg/mL PR-ASG or no PR-ASG. The treated cells were washed with Krebs Ringer buffer. They were then cultured for one day with Krebs Ringer buffer containing 0.1 mM glucose supplemented with IGF-1 (0–300 ng/mL) and PR-ASG (▪: 4 µg/mL or □: 0 µg/mL). The glucose utilized was less than 1.0 nmol/hr/10^6^ cells at zero concentration of IGF-1. E) Cells were pre-washed with Krebs Ringer buffer, followed by exposure for one day to PR-ASG (▪: 4 µg/mL or □: 0 µg/mL) plus Krebs Ringer buffer containing 0.1 mM glucose/300 ng/mL IGF-1 and increasing amounts of 2-DG (0–10 mM). In B), D), and E), glucose utilization was examined in the cell supernatants using a glucose assay kit. Values were expressed as means ± SEM, n = 6 individual experiments and were analyzed by two-way ANOVA followed by Bonferroni's post test. The singular and multiple asterisks denote a statistically significant difference between non-shaded and shaded columns at the same concentration of IGF-1 treatment (B, D) or 2-DG-treatment (E) (*p<0.05, **p<0.01).

The effect of PR-ASG on IGF-1 production was examined using INS-1 cells. As shown in Fig. 2A, IGF-1 secretion did not occur in STZ-negative medium (first non-shaded column), but addition of PR-ASG to the STZ-negative medium stimulated IGF-1 secretion in a dose-dependent manner (0.4 to 8 µg/mL), increasing up to more than 500 pg/mL of IGF-1. On the contrary, very little IGF-1 was secreted for STZ-treated cells at any concentration of PR-ASG presumably because STZ induced cell apoptosis.

To confirm the effect of PR-ASG on IGF-1 expression in INS-1 cells, mRNA expression was analyzed by RT-PCR. The mRNA expression of IGF-1 was induced in the presence of serum. Addition of PR-ASG revealed increased IGF-1 expression by INS-1 cells after they were cultured with 4 and 8 µg/mL of PR-ASG (Fig. 2C).

### Insulin secretory response in INS-1 cells

When compared to a low glucose (2 mM) KRB buffer (1.4±0.2 ng/mL per 1-hr culturing, mean ±SEM), a high glucose (12 mM) KRB buffer produced a 8.5 –fold increase in glucose-stimulated insulin secretion by INS-1 cells. PR-ASG did not alter insulin secretion in the range of 0.4–40 µg/mL (data not shown).

### IGF-1-dependent glucose utilization in INS-1 cells: glycolytic pathway

The relevance of PR-ASG to IGF-1 in the glycolytic pathway was ascertained by examining glucose utilization by INS-1 cells. IGF-1-dependent glucose utilization via glycolysis including uptake and consumption of extracellular glucose, was examined by assessing the amount of glucose remaining in the cell-culture medium. The glucose utilization by INS-1 cells was very low at zero concentration of IGF-1, and showed less than 1.0 nmol glucose/hr/10^6^ cells. A higher range of IGF-1 treatment (10 to 300 ng/mL), however, produced dose-dependent glucose utilization (Fig. 2B) with more than 70% (11.7 nmol glucose/hr/10^6^ cells) of glucose consumed after exposure to 200–300 ng/mL of IGF-1. Additionally, IGF-1-related glucose utilization was observed at 3 picogram levels of IGF-1 (8.5±2.1% for 200 pg/mL, 15.5±3.1% for 500 pg/mL, 18.0±3.1% for 1000 pg/mL, n = 6, respectively), corresponding to levels of IGF-1 secretion induced by PR-ASG as shown in Fig. 2A. The presence of PR-ASG showed a synergic effect on glucose utilization in relation to exogenously added IGF-1, most significantly at 10 and 50 ng/mL(**p<0.01), and 100 ng/mL(*p<0.05) of IGF-1. More than 80% utilization of glucose was observed at 200–300 ng/mL of IGF-1.

STZ treatment suppressed the INS-1 cell response to IGF-1, and decreased glucose consumption down to 50% at 300 ng/mL of IGF-1 (Fig. 2D).

PR-ASG, however, protected cells from the negative effects of STZ, showing statistically significant enhancement of glucose utilization in response to IGF-1(**p<0.01 in 100 and 200 ng/mL of IGF-1).

Treatment with 2-DG (0–10 mM) also inhibited the effect of IGF-1 (300 ng/mL) on the INS-1 cells (Fig. 2E). Again, addition of PR-ASG was observed to ameliorate and reverse the inhibition of glucose utilization by 2-DG in a statistically significant manner on the glyocolytic pathway (**p<0.01 in 1 and 5 mM 2-DG treatment) (Fig. 2E).

### Anti-oxidative stress by G6PD activity in INS-1 cells: pentose phosphate pathway

The effect of PR-ASG on the pentose phosphate pathway was examined by measuring G6PD activity in the cells. Non-treated cells showed G6PD activity of 850 nmol formazan/hr/10^6^ cells, which was reduced to 550 nmol formazan/hr/10^6^ cells by STZ treatment.

**Figure 3:**
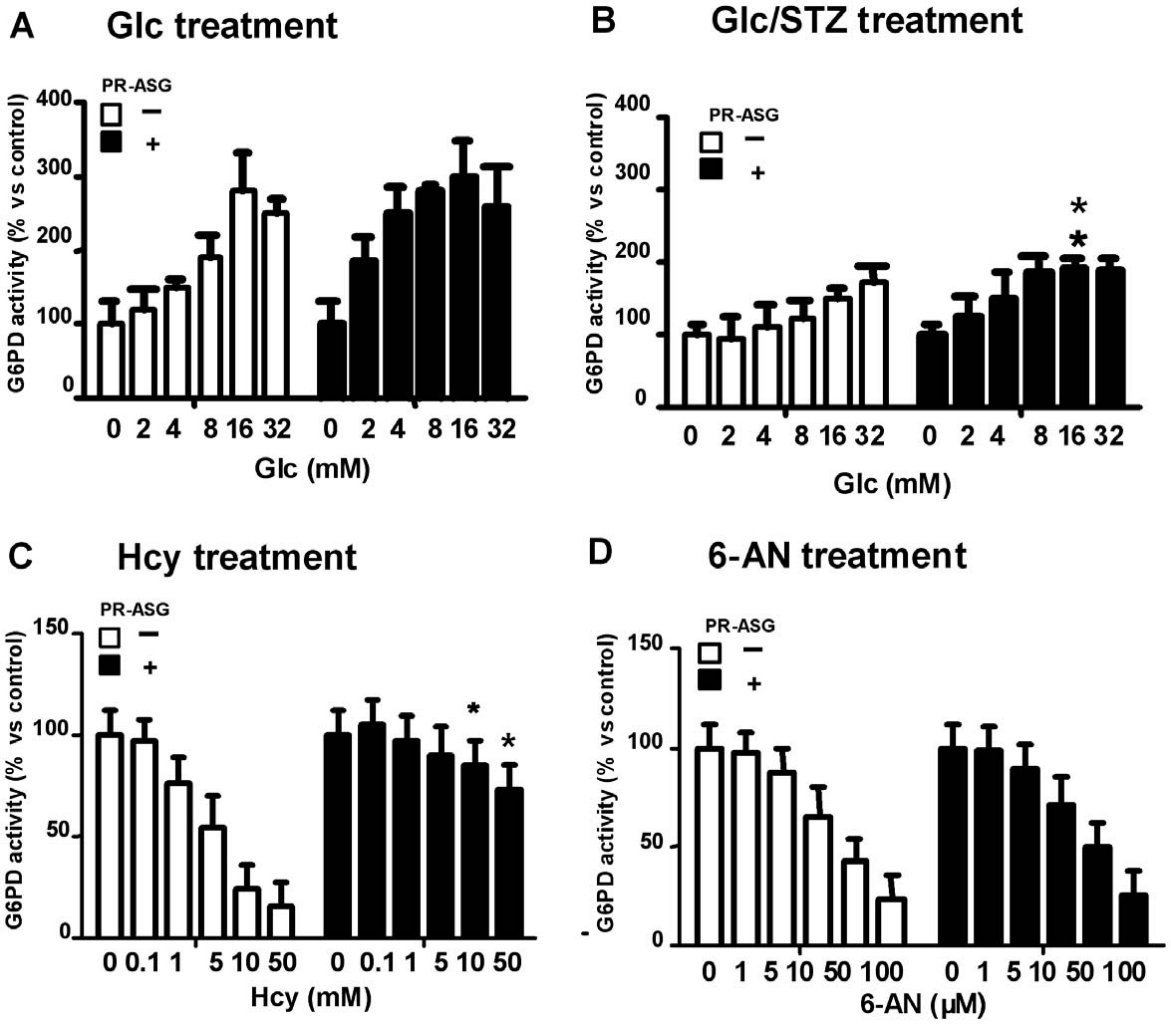
Effect of PR-ASG on G6PD activity in the pentose phosphate pathway in INS-1 cells. A) Cells were treated for 30 min at 37°C with serum-free RPMI-1640 medium containing glucose (0–32 mM) and PR-ASG (▪; 4 µg/mL or □; 0 µg/mL). Subsequently, cells were trypsinized and G6PD activity in the cell lysate was measured, as described in Materials and Methods. G6PD activity is expressed as a percent value, with 100% equals to 850 nmol formazan/hr/10^6^ cells. B) Cells were exposed for 1 hr to 1 mM STZ in RPMI1640 medium containing either 4 (▪) or 0 (□) µg/mL of PR-ASG. Subsequently, cells were treated for 30 min at 37°C with serum-free RPMI-1640 medium containing increasing amounts of glucose (0–32 mM) and either 4 (▪) or 0 (□) µg/mL of PR-ASG. The cells were then harvested, and the cell lysate was measured for G6PD activity, as described in Materials and Methods section. In the condition of STZ-treatment, G6PD activity is expressed as a percent value, with 100% equal o 550 nmol formazan/hr/10^6^ cells. C) Cells were exposed for 24 hr to increasing amounts of Hcy (0–50 mM) in RPMI medium without folic acid and containing either 4 (▪) or 0(□) µg/mL of PR-ASG. Cultured cells were harvested, and assessed for G6PD activity, as described in Materials and Methods. G6PD activity is expressed as a percent value, with 100% equal to 850 nmol formazan/hr/10^6^ cells. D) Cells were exposed for 24 hr to increasing amounts of 6-AN (0–100 µM) in RPMI1640 medium containing either 4 (▪) or 0 (□) µg/mL of PR-ASG. The cells were then harvested and assessed for G6PD activity, as described in Materials and Methods. G6PD activity is expressed as a percent value, with 100% equal to 850 nmol formazan/hr/10^6^ cells. In A), B), C), and D), values were presented as means ± SEM, n = 6 individual experiments and are analyzed by two-way ANOVA followed by Bonferroni's post test. The single and multiple asterisks denote a statistically significant difference between non-shaded and shaded columns at same concentration of Glc treatment (A, B), Hcy treatment (C), or 6-AN treatment (D) (*p<0.05, **p<0.01).

The intracellular G6PD activity responded to changes in extracellular glucose concentration, ranging from 2–32 mM (Fig. 3A). Non-STZ-treated cells did not show a remarkable enhancement of the pentose phosphate pathway, even with addition of PR-ASG (4 µg/mL) (Fig. 3A). Although STZ-treated cells showed relatively attenuated G6PD activity (550 nmol formazan/hr/10^6^ cells), mild enhancement of this activity was observed in response to changes in extracellular glucose concentration; furthermore, PR-ASG treatment of these cells showed a further, statistically significant increase of G6PD activity at 16 mM Glc (**p<0.01, Fig. 3B).

Treatment of the cells with Hcy produced a decrease in G6PD activity (Fig. 3C). Again, PR-ASG reversed this influence of Hcy on G6PD activity, with a statistical difference (**p<0.01 in 10 mM Hcy treatment, *p<0.05 in 50 mM Hcy treatment, Fig. 3C), suggesting normalization of the pentose phosphate pathway. However, these beneficial effects of PR-ASG were eliminated with 10–100 mM 6-AN treatment, inhibiting G6PD activity regardless of PR-ASG concentration with no statistical difference (Fig. 3D).

### Oxidative stress and anti-oxidative stress in INS-1 cells: ROS level and HTase activity

**Figure 4:**
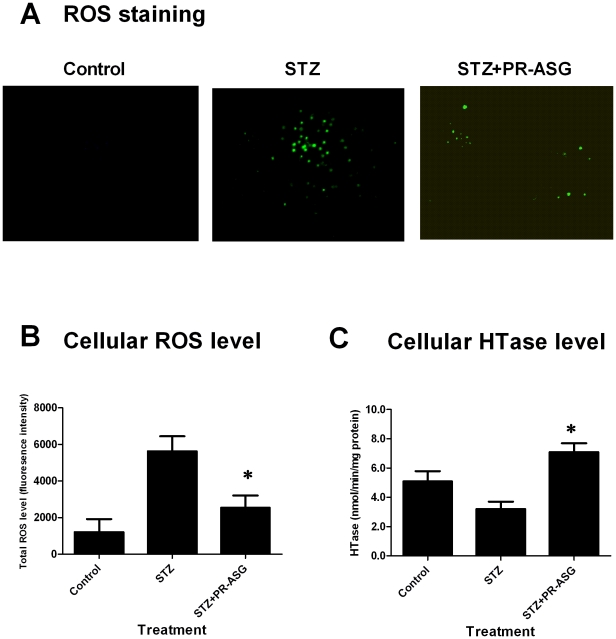
Effect of PR-ASG treatment on cellular ROS level and HTase activity in STZ-exposed cells. A) Initially, cells were incubated under one of these sets of experimental parameters: STZ-negative, PR-ASG-negative (control); 1 mM STZ (STZ group); or 1 mM STZ supplemented with 4 µg/mL of PR-ASG (STZ+PR-ASG). After 1 hr of incubation, the cells were pre-washed with fresh medium, stained with DCF-DA (1.0 µM), and incubated in darkness for 10 min. DCF-DA green fluorescence was viewed under a fluorescence microscope. B) DCF-DA green fluorescing cells were also quantitated by a Perkin-Elmer Victor V multilabel plate reader with excitation/emission filters set at 490 and 535 nm, and expressed as the percent of the total number of cells that exhibit DCA-DA green fluorescence. C) Cells (2.5×10^5^ in 1 mL of 10% serum- containing RPMI 1640 medium well) were cultured in12-well plates. One day later, the cells were divided into 3 groups fro 1 hour of exposure to one of the following in RPMI medium containing 1.0 µg/mL rat LDL; no STZ with no PR-ASG (control); 1 mM STZ (STZ); or 1 mM STZ with 4 µg/mL of PR-ASG (STZ+PR-ASG). Subsequently, cells were pre-washed; cultured for 24 hours in serum-free RPMI-1640 medium with 0 or 4 µg/mL of PR-ASG; harvested; and eventually used for enzyme preparation. The cellular supernatant produced during enzyme preparation was assessed for activity using an Alfresa auto HTLase assay kit (Alfresa Pharma Corp., Osaka, Japan). One unit of HTase activity is defined as 1 nmol of HT hydrolyzed in 1 min by 1 mg of protein. In B) and C), values were expressed as means ± SEM, n = 6 individual experiments, and analyzed by one-way ANOVA followed by Tukey's multiples comparison test and then Dunnet's test. The single asterisk denotes a statistically significant difference between STZ and STZ+PR-ASG (*p<0.01).

Cells were treated with STZ, and assessed for PR-ASG's anti-oxidative action. ROS staining demonstrated that STZ treatment caused a highly elevated total ROS level in cells (Fig. 4A and B). On the other hand, PR-ASG treatment reversed this effect and decreased ROS production in cells by a statistical difference (*p<0.01).

Under the condition of STZ-induced oxidative stress in the absence of PR-ASG, cellular HTase activity was slightly decreased, but not statistically different from the control group (Fig. 4C). In the presence of PR-ASG, however, cellular HTase activity showed statistically significant (*p<0.01) elevation in STZ-treated cells in STZ-stressed under condition of oxidative stress.

## Discussion

PR-ASG is a unique ingredient found in the bran of pre-germinated brown rice that can be purified from the glycolipid fractions, as previously reported [5]. In our current study, PR-diet was tested in STZ-induced diabetic rats. Compared with diabetic rats fed control diets, the PR-diet fed rats showed an improvement of serum metabolic parameters including serum insulin levels and peripheral NCV. This activity was considered to be derived from PR-ASG, because of pancreatic IGF-1 elevation found in the diabetic rats fed PR-diet. In addition, we confirmed that PR-ASG shows an IGF-1-inducing effect on the pancreatic β-cell line, INS-1 (834/40). Our cells demonstrated glucose-stimulated insulin secretion, but not PR-ASG-stimulated insulin secretion. We showed that this is a glucose-sensitive cell line that possesses the potential ability to produce and release IGF-1 into the culture medium [21].

STZ shows selective cytotoxicity on pancreatic β-cells, and thus causes insulin-deficient diabetes. It has been suggested that STZ may generate Reactive oxygen species (ROS) such as nitric oxide (NO) and superoxide (O_2_^−^), thereby inducing apoptosis of pancreatic β-cells [22], [23], [24]. In our current study, PR-ASG showed a protective effect similar to that of serum against STZ-induced apoptosis (Fig. 1C). We are not sure whether this effect of PR-ASG on STZ-treated INS-1 cells involves a cell proliferation mechanism, as the growth factors are found in both serum and tissues. We do however, report two significant findings of our current study: 1) that pancreatic IGF-1 levels closer to normal level, as compared with liver and serum levels, are remarkably elevated in STZ-diabetic rats fed PR-ASG in an animal study (Table 1); and 2) that PR-ASG-treated INS-1 cells produce and release IGF-1 into the culture medium in our *in vitro* study (Fig. 2A and C). Addition of serum to the medium resulted in IGF-1 mRNA expression by the INS-1 cells. This suggests that PR-ASG is also related to cell proliferation via IGF-1 production and secretion; although a remarkable level of cell proliferation has not yet been observed according to PR-ASG treatment, as compared with the remarkable cell proliferation level seen secondary to serum treatment.

**Figure 5:**
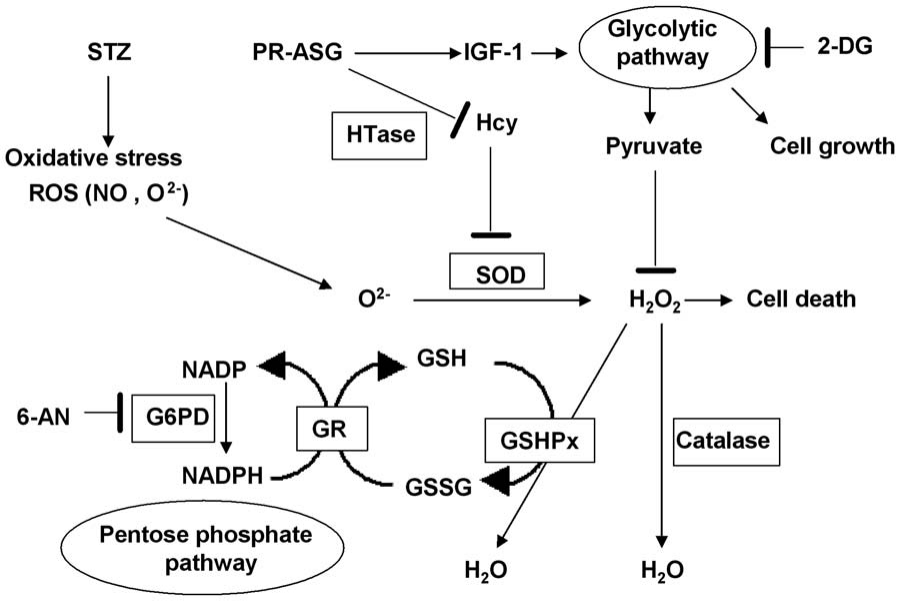
Suggested mechanism for PR-ASG's protective action against oxidative stress in INS-1 cells. Reactive oxgen species (ROS) generated from STZ exposure are metabolized and inactivated by SOD, to produce H_2_O_2_. It has been shown that an imbalance in the coordinated expression/activity of glutathione peroxidase (GSHPx) and glutathione reductase (GR) can cause excessive generation of ROS, leading to oxidative stress. GSHPx converts H_2_O_2_ to water using glutathione (GSH). Maintenance of the redox state in cells' redox state is controlled by intracellular regulators such as reduced glutathione (GSH) and NADPH. Both GR and glucose-6-phosphate dehydrogenase (G6PD) are enzymes suspected to have protective activity against conditions of oxidative stress. On the other hand, 2-deoxy-glucose (2-DG, an inhibitor of glycolytic pathway) and 6-aminonicotinamide (6-AN, an inhibitor of pentose phosphate pathway) both decrease the cellular levels of pyruvate and NADPH. This leads to an accumulation of H_2_O_2_, the building of which acts to induce apoptosis. PR-ASG's defensive action is exerted via an increase in two targets: increasing homocysteine-thiolactonase (HTase) enhances homocysteine (Hcy) metabolism; and increasing pyruvate enhances protection against cell via IGF-1-related glucose metabolism.

The primary defense system against oxidative stress in β-cells is controlled by superoxide dismutase (SOD), glutathione peroxidase (GSHPx), and catalase, which act to control ROS levels during oxidative stress (Fig. 5). Maintenance of the redox status in cells is controlled by intracellular regulators such as reduced glutathione (GSH) and NADPH. This mechanism involves two enzymes: glutathione reductase (GR) and glucose-6-phosphate dehydrogenase (G6PD). The overall effect of the antioxidant system depends on the intraceullar balance between these antioxidant enzymes [6], as a critical balance exists in the β-cells between endogenous ROS generation and antioxidant defense. As expected, PR-ASG showed an IGF-1-induced enhancement of glycolytic pathway in order to overcome much of the STZ-induced oxidative stress (Fig. 2D).

In the antioxidant enzyme system, SOD's role is to inactivate ROS by catalyzing the O_2_^−^ to H_2_O_2_, which is then further metabolized to H_2_O and O_2_ by catalase and GSHPx (Fig. 5). We demonstrated ROS elevation in the INS-1 cell by STZ-induced-oxidative stress and ROS suppression by PR-ASG treatment (Fig. 4A and B).

Extracellular SOD (EC-SOD) is sensitive to Hcy treatment. Hcy subjects cells to additional oxidative stress by inhibiting the binding of EC-SOD in fibroblasts [25] and endothelial cells [26].

Considering the mechanism by which PR-ASG effects pancreatic β-cells, it is suggested that HTase-related antioxidant control is more important than ordinary defense for protection of EC-SOD against the oxidative stress of Hcy. We also demonstrated a change in HTase activity after PR-ASG treatment (Fig. 4C).

IGF-1 is ubiquitously distributed in various tissues and cells, and it plays an important role for prevention of insulin resistance and protection against age-related oxidative damage, leading to β-cell apoptosis. In our study, the effect of STZ-induced diabetes has been observed as a decrease in IGF-1 levels [27] (Table 1). The glucose-dependent IGF-1 activation system is closely coupled to glucose metabolism, glycolytic pathway, and the pentose phosphate pathway. The importance of G6PD activity in the pentose phosphate pathway is to maintain the cellular ratio of NADPH/NADP, and to up-regulate G6PD activity when required for the cell apoptotic response to ROS [7], [8]. The glycolytic pathway is enhanced for cell proliferation subsequent to activation of the glucose-dependent IGF-1 activation system [8]. IGF-1 is produced and secreted by INS-1 cells secondary to treatment with PR-ASG, although this mechanism is still unknown. Pyruvate, a metabolite of the glycolytic pathway, is known to lessen oxidative stress by scavenging H_2_O_2_ [28]. We have demonstrated that PR-ASG affects IGF-1-induced-activation of glycolysis as the IGF-1-dependent glucose utilization is blocked by 2-DG, a specific inhibitor of glycolytic pathway (Fig. 2E). On the contrary, none of the direct targets affected by PR-ASG were involved in the pentose phosphate pathway. We also found that PR-ASG could not ameliorate the effects of the pentose phosphate pathway inhibitor, 6-AN (Fig. 3D). It has been reported that hyperhomocysteinemia led to significant lowering of G6PD activity [29]. It therefore follows that Hcy treatment of INS-1 cells produced a decrease in G6PD activity (Fig. 3C), presumably by a protein homocystinylation mechanism. Subsequent PR-ASG treatment of Hcy-stressed cells showed an ameliorating decrease of G6PD activity by Hcy treatment, indirectly regulating the pentose phosphate pathway. We also demonstrated cellular HTase activity was decreased by STZ-stress and recovered by PR-ASG (Fig. 4C).

In summary, our findings suggest that the PR-diet markedly reduced hyperglycemia and peripheral neuropathy in STZ-induced diabetic rats; and that this reduction was due to increased IGF-1 secretion and anti-oxidative stress enzyme HTase activation. The animal results supported our other evidence in showing that PR-ASG acts on INS-1 cells through both an IGF-1-dependent mechanism, and an HTase activation mechanism of ameliorating decreased glycolysis activity in oxidatively stressed cells.

It is therefore collectively suggested that PR-ASG may protect pancreatic β-cells via enhancement of an IGF-1 dependent autocrine system, leading to subsequent activation of an IGF-1 dependent–defense system, which, together with HTase activation, may be useful in the treatment of oxidative stress and its complications in diabetes.
